# Altered Peroxisome-Proliferator Activated Receptors Expression in Human Endometrial Cancer

**DOI:** 10.1155/2012/471524

**Published:** 2012-02-19

**Authors:** Paweł Knapp, Adrian Chabowski, Agnieszka Błachnio-Zabielska, Katarzyna Jarząbek, Sławomir Wołczyński

**Affiliations:** ^1^Department of Gynecology, Medical University of Bialystok, 15-222 Bialystok, Poland; ^2^Department of Physiology, Medical University of Bialystok, 15-222 Bialystok, Poland; ^3^Department of Gynecological Endocrinology, Medical University of Bialystok, 15-276 Bialystok, Poland

## Abstract

Peroxisome proliferator-activated receptors (PPARs) belong to a family of nuclear hormone receptors acting as transcriptional factors, recently involved also in carcinogenesis. Present study was undertaken to evaluate the presence and subcellular localization of different PPAR isoforms (**α**, **β**, **γ**) in healthy endometrial tissue (*n* = 10) and endometrial carcinoma (FIGO I, endometrioides type, G1, *n* = 35). We sought to analyze PPARs mRNA content as well as protein immunohistochemical expression that was further quantified by Western Blot technique. For both PPAR**α** and PPAR**β**, protein expression was significantly higher in endometrial cancers compared to normal endometrial mucosa. In opposite, PPAR**γ** protein expression was lower in endometrial cancer cells. In each case, immunohistochemical reaction was confined to the perinuclear and/or nuclear region. At the transcriptional level, the content of mRNA of all PPAR subunits did not follow the protein pattern of changes. These results provide evidence for altered PPAR's protein expression and disregulation of posttranslational processes in endometrial cancers.

## 1. Introduction

Peroxisome proliferator-activated receptors (PPARs) are ligand-activated transcription factors of the nuclear hormone receptor superfamily [1, 2]. Three distinct PPAR isoforms termed *α*, *β*/*δ*, and *γ* have been identified [3–5]. They share several structural common features, but each is distinctly expressed in different tissues. PPAR*α* and *γ* are predominantly expressed in heart, muscles, liver, and in adipocytes [3–5]. PPAR*β* is more ubiquitously expressed, but shares certain common downstream effects with PPAR*α* [4, 5]. For both PPAR*α* and *β*, it has been shown that their activation is responsible for the enhancements in energy substrate utilization [4]. This ability of PPARs to regulate cellular metabolism leads to the question, whether the tumor cells have altered PPAR expression. It is tempting to speculate so, since high energy substrate consumption is a well-known feature of neoplastic cells, especially these with high rates of cell proliferation. Indeed, a growing number of researches begin to suggest an important role of PPAR activation in the biology of the neoplastic process. Furthermore, some studies offer the prospect of using PPAR as a destination point of action for both prevention and treatment of cancers [6–9].

Endometrial cancer (EC) is one of the most widespread gynecologic cancers in Europe, and according to FIGO classification (International Federation of Gynecology and Obstetrics) as well as Bokhman theory [10] endometrial cancer—endometrioid type (FIGO stage I, type I) is the most frequent. In addition, EC is usually present with well-differentiated morphology (G1) along with an endometrioid features [10]. Based on that we sought to investigate PPARs expression in this type of cancer. We examined different PPAR (*α*, *β*/*δ*,*γ*) isoforms expression at the level of transcription (mRNA) and proteins (by immunohistochemistry and Western Blot technique).

## 2. Material and Methods

The present study conforms with the principles outlined in the Declaration of Helsinki and was approved by the Ethical Committee for Human Studies of the Medical University of Bialystok. The patients suspected to have the cancer of corpus uteri were examined in outpatient clinic, and biopsies were taken for routine histopathological examination. Standard histopathological parameters were determined by two independent pathologists. Based on this evaluation the two groups of women were included for the analysis: (a) patients with endometrial cancer (*n* = 35) and (b) patients with normal endometrial tissues (control group, *n* = 10). In the group of diagnosed EC, cases with type endometrioid, FIGO I, grade 1, were included in the study. Control endometrial tissue was gathered during nononcological operations, mostly because of fibroids. In each case, endometrial cancer risk factors such as age, the presence of hypertension, obesity, and type 2 diabetes were evaluated.

### 2.1. RNA Extraction and cDNA Synthesis

Total RNA was extracted from frozen endometrial malignant and normal tissues according to Chomczynski and Sacchi method [11]. RNA integrity was verified by elecrophoresis in 1.5% agarose gel and staining with ethidium bromide, and by amplification of housekeeping gene, 18s rRNA, 1 *μ*g of total RNA was used to prepare cDNA. cDNA synthesis was performed in 50 mM Tris-HCL (pH 8.3), 75 mM KCl, 3 mM MgCl_2_, 10 mM DTT 1 mM dNTP mix (Promega), 2.5 *μ*M oligo dT_15_, 20 U RNasin Ribonuclease Inhibitor (Promega), and 100 U MMLV Reverse Transcriptase (Promega) in a final volume of 40 *μ*l using MJ Research Thermal Cycler (Model PTC-200, Watertown, Massachusetts, USA). For reverse transcription, the mixtures were incubated at 42°C for 60 min and then heated at 95°C for 5 min and finally rapidly cooled at 4°C. To determine the mRNA level of PPARs we used Assays-on-Demand Gene Expression Assay Mix (Applied Biosystems). All real-time PCR reactions were performed using ABI Prism 7000 Sequence Detection System (Perkin-Elmer Applied Biosystems, USA). For each PCR run, a master mix was prepared with 10 *μ*l 2x Taq Man Universal PCR Master Mix (Applied Biosystems), 1 *μ*l 20x Assays-on-Demand Gene Expression Assay Mix (Applied Biosystems), 5 *μ*l cDNA, and sterile water to final volume of 20 *μ*l. The relative quantification was given by the ratio between the mean value of the target gene and the mean value of the reference gene for each sample. PCR products were obtained by amplification of cDNA from normal endometrial tissue using specific primers as follows: sense: 5′-CGA GGC CGG CGA TCT AG-3′; antisense: 5′-ACG CGG GGA CTC CGT AAT G-3′ for PPAR-*α* sense: 5′-CAT GGA GCA GCC ACA GGA G-3′, antisense: 5′-TGC ATG AAC ACC GTA GTG GAA G-3′ for PPAR-*β*: sense: 5′-CAA GGC TTC ATG ACA AGG GAG-3′, antisense: 5′-CGT GTT CCG TGA CAA TCT GTC T-3′ for PPAR-*γ*. PCR was carried out in final volume of 50 *μ*l using 25 pmol of each of the primers, 40 *μ*M of each of dNTPs, 1.5 U Taq polymerase (Finnzymes, Finland), 5 *μ*l 10-fold PCR buffer, and 5 *μ*l cDNA. PCR was carried out under the following conditions: 5 min at 95°C, 1 min denaturation at 95°C, 1 min annealing at 60°C, 1 min extension at 72°C for 40 cycles, with an additional 10 min extension for the last cycle. Amplified products were separated on a 2% (w/v) agarose gel, extracted and purified from agarose slices using DNA Gel Extraction Kit (Millipore, USA), quantified by the use of One Dscan/Zero Dscan software (Scanalytics Inc., USA), and then diluted in sterile water [12].

### 2.2. Western Blot Analysis

Tissue samples (control and endometrial cancer) were homogenized in RIPA buffer (1 : 10 v/w, ice-cold, pH 7,4), with the addition of protease inhibitors cocktail (1 mM EDTA, 1 mM PMSF, 1 *μ*g/mL aprotinin, 1 *μ*g/mL leupeptin, 1 *μ*g/mL pepstatin). Then samples were centrifuged at 10,000 g for 30 min at 4°C and the supernatant was analyzed further. Protein content was measured with the BCA protein assay kit (Sigma). Bovine serum albumin was used as a standard. Proteins (50 *μ*g) were separated by SDS PAGE on 10% gel. Separated proteins were transferred on nitrocellulose membranes (BioRad) in transfer buffer (25 mM Tris-HCl, pH 8.3, 192 mM glycine, 20% methanol) at 14 V in 4°C. An equal sample loading was confirmed by Ponceau S stain. Then nitrocellulose blots were placed in blocking buffer (5% nonfat milk in TBS-T) for 1 h. The membranes were incubated with primary antibodies against PPAR*α*, PPAR*β*, PPAR*γ* (ab8934, ab23673, ab19481 Abcam, UK) or *β*-actin (ab3280, Abcam, UK), for 2 h at 4°C. After three washings in TBS-T, membranes were incubated with an alkaline phosphatase-conjugated secondary antibody (Sigma). Protein bands were scanned and quantified using a Gel Doc EQ system (Bio-Rad). The total content of PPARs in homogenate was normalized to the *β*-actin expression and presented in arbitrary units (ODU-optical density units).

### 2.3. Immunohistochemical Tissue Staining

For immunohistochemical studies 2-3 representative sections from each case of the endometrial cancer and normal tissues were selected. PPARs immunoexpression was evaluated by the use of the polyclonal antibody (Santa Cruz Biotechnology, USA) as recommended by the producer. Briefly, the sections were deparaffinized in xylenes and hydrated through graded alcohols. Antigen unmasking was performed using heat treatment in a microwave oven at 750 W for 7 minutes in a container with 10 mM sodium citrate buffer, pH 6.0. Sections were allowed to cool in the buffer at room temperature for 30 minutes and were rinsed in deionized H_2_0 three times for 2 minutes each. The endogenous peroxidase activity was blocked with 1% hydrogen peroxide for 20 minutes. After rinsing in PBS, the sections were incubated for 1 hour with 1.5% normal blocking serum in PBS. The blocking reagent was removed, and then the sections were incubated with primary antibody at 4°C overnight using staining chamber (The Binding Site, United Kingdom). Primary antibodies were diluted in PBS with 1.5% normal blocking serum. Omitting primary antibodies served as negative control. After rinsing in three changes of PBS, a streptavidin-biotin-peroxidase complex technique was used to reveal antibody-antigen reactions (EnVision kit, Dako, Denmark). Staining was routinely developed using 3,3′-diaminobenzidine as a chromogen (Dako, Denmark). Sections were counterstained with hematoxylin. Two independent pathologists, who were blinded to the clinicopathological data of the patients, evaluated immunostainings with the use of light microscopy (20x and 40x objectives). The evaluation of PPAR expression was analyzed in 10 different tumor fields, and the mean percentage of tumor cells displaying positive staining was scored. No staining of the cells was observed in any of the tumor sections after omitting the first antibody. Immunohistochemical expression for each PPAR isoform is presented as the percentage (%) of the immunopositive cells present in healthy endometrium or endometrial cancer tissue.

### 2.4. Statistical Analysis

Statistical comparisons were made by using appropriate tests (Mann-Whitney *U* test, *t*-Student test, and/or one-way ANOVA followed by the Newman-Keul post hoc test). *P* < 0.05 was considered statistically significant.

## 3. Results

All selected patients (*n* = 10) in the control group with normal endometrial mucosa had no hypertension or diabetes nor obesity (BMI was 28,2), with the average age around 48,5 (range 35–54). The average age in the group of patients with endometrial cancer was around 59 (range 43–73), and there was no evidence either of obesity (BMI was 30,8) or hypertension nor diabetes (data not shown).

### 3.1. Immunohistochemical Expression

**Figure 1:**
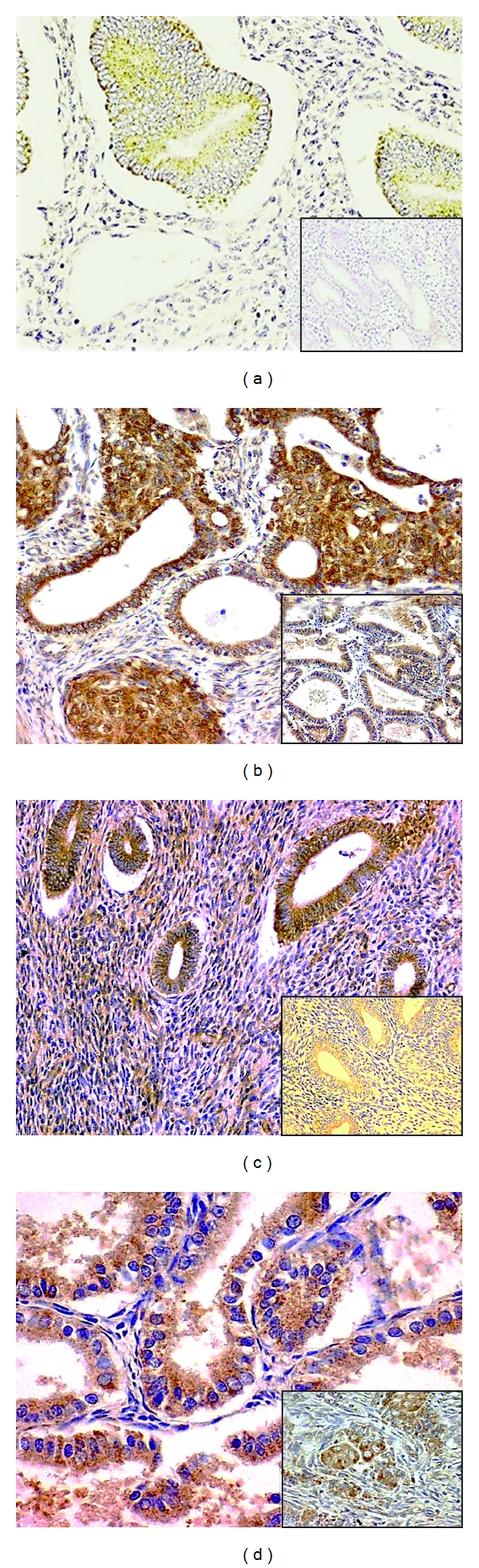
Immunohistochemical expression of PPAR*α* in normal endometrium (a) and endometrial cancer (b) and PPAR*γ* in normal endometrium (c) and endometrial cancer (d). Some cells show enhanced accumulation of PPARs in nuclear and perinuclear area.

Positive PPAR (-*α*, -*β*, -*γ*) immunohistochemical staining was detected in normal endometrial mucosa (for PPAR *α*: 57%, PPAR *β*: 55%, and PPAR*γ*: 61%) as well as in endometrial cancers (for PPAR*α*: 78%; PPAR*β*: 77%; PPAR*γ*: 84%) (Figure 1). With respect to intensity of the staining, we observed more frequently a stronger positive reaction in cancer cells than in healthy mucosa, but not in all cases (data not shown). A trend towards higher expression of PPAR *α*, *β* in endometrial cancers was noticed (+21% and +22%, *P* = 0.067, resp.). In opposite, EC cells showed lower expression for PPAR*γ* (−23%, *P* < 0.05). Localization of the staining was similar for all the PPARs isoforms. Immunopositive cells in normal and endometrial cancer tissue were found broadly in nuclear and perinuclear region (Figure 1).

### 3.2. PPARs mRNA Content

The mRNA content of all PPARs was examined in normal endometrial mucosa (*n* = 10) as well as in endometrial cancers (*n* = 35). The mRNA expression of each PPAR isoform was significantly higher in normal endometrial tissue comparing with EC (PPAR*α*:+ 3,1–fold; PPAR*β*: +3,8-fold; PPAR*γ*: +4,1-fold; *P* < 0.05; Table 1).

### 3.3. PPARs Protein Expression

**Figure 2:**
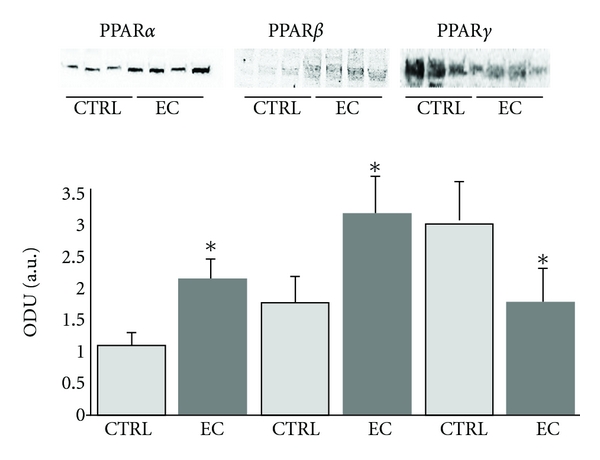
The expression of PPARs (*α*, *β*, *γ*) proteins (optical density units (ODU)) in normal and endometrial cancer tissues. *P < 0.05 (CTRL); normal endometrium versus endometrial cancer (EC).

Western blots analyses confirmed greater immunohistochemical expression of PPAR*α* and PPAR*β* isoforms in endometrial cancer tissue comparing with normal mucosa (PPAR*α*: +0,7-fold; PPAR*β*: +2,0-fold; *P* < 0.05; Figure 2). An opposite effect was observed for the expression of PPAR*γ*, which was significantly lower in EC (PPAR*γ*: −1,5-fold; *P* < 0.05; Figure 2).

## 4. Discussion

The present study was undertaken to characterize the expression of PPARs in endometrial cancers (EC) at the transcriptional (mRNA) and posttranscriptional (proteins) levels. Immunohistochemistry was applied for the evaluation of the PPARs immunoexpression and subcellular distribution. Protein expression was further quantified by Western Blot technique. To the best of our knowledge, this is the first study to report that only the expression of PPAR*α* and *β* is relatively higher in EC, but not PPAR*γ*. The disassociation of mRNA content and respective protein product were also found in endometrial cancers. This discrepancy of the mRNA content and the expression of respective protein is commonly observed in neoplastic tissues [12, 13].

### 4.1. PPAR*γ* Expression

We found reduced immunohistochemical PPAR*γ* expression in EC, which is consistent with other reports showing rather moderate immunoreactivity of PPAR*γ* expression in endometrial carcinoma cells [14, 15]. This relatively low PPAR*γ* expression was also found in other tumor cells, and some studies begin to suggest that PPAR*γ* agonists may inhibit cell proliferation in the neoplastic cell lines [16–18]. It seems highly possible, since several in vitro studies have revealed that pioglitazone, a PPAR-*γ* agonist, induces cell differentiation [19] and several clinical studies have demonstrated that the activation PPAR-*γ* increases the degree of histopathological differentiation of liposarcoma [19, 20]. However, it is not the case for all neoplastic transformation, as others demonstrated that highly malignant cancer cell lines are characterized by higher expression of PPAR*γ* and these data suggest that PPAR*γ* may act in a cancer-permissive fashion [21, 22].

### 4.2. PPAR*α* and/or PPAR*β* Expression

In our study we found increased expression (immunoreactivity further quantified by Western Blot) of both PPAR*α* and PPAR*β* isoforms in EC compared to healthy endometrium. This finding may imply a possible role for both PPARs (*α* and *β*) in neoplastic transformation of endometrial cells. However, we are aware of the limitations of our study. First the study was limited to one type of endometrial cancers (FIGO 1), and an open question remains whether there is a progression of the PPARs expression in more advanced endometrial cancers. Secondly, some reports suggest that there is a considerable background immunoreactivity when PPARs expression is measured by immunohistochemistry [23, 24]. This is important as there are a number of reports showing either an increase or decrease in PPAR (*α* and *β*) immunohistochemical expression, even in the same tissue [23, 25]. Nonetheless, the speculations concerning PPAR*α*/*β* relative expression and function in cancer cells are further based on their opposite to PPAR*γ* physiological roles. PPAR*γ* is thought to be primarily involved in processes that augment differentiation of the cells and/or storage of energy, and PPARs*α*/*β* activation presumably enhances the processes related to the fuel expenditure. This suggests a possibility that neoplastic cells may have greater PPAR*α*/*β* expression/activity, which should activate genes controlling cellular metabolism and result in faster metabolic rates of cancer cells. From rodent studies it is becoming evident that chronic treatment with PPAR*α* agonist induces incidences of liver tumors through a mechanism, that results in an increase of both cellular proliferation and oxidative stress [26]. However, such a tumorigenic influence of PPAR*α* activation was found only in animal studies. As far there is no evidence that PPAR*α* agonists such as fibrates are associated with elevated risk of cancer in humans [27, 28].

 In summary, we provide evidence for altered expression of different PPAR isoforms in endometrial cancer cells, namely, greater expression of PPAR*α* and PPAR*β*, with concomitant reduction of PPAR*γ* in EC.

## Figures and Tables

**Table 1:** Expression of PPARs mRNA in normal and endometrial cancer tissues (*Mann-Withney test).

Parameters (fmol/*μ*g of RNA)	Normal endometrium *n* = 10			Endometrial cancer *n* = 35			
	Median	Low quartile	High quartile	Median	Low quartile	High quartile	P*
PPAR*α*	3,136	0,77	7,113	0,986	0,5507	1,783	*P* < 0.05
PPAR*β*	5,373	4,121	11,64	1,417	0,778	2,831	*P* < 0.0001
PPAR*γ*	7,148	5,568	19,77	1,721	0,601	4,883	*P* < 0.0001
